# A rare case report of small bowel leiomyosarcoma with literature review

**DOI:** 10.1093/jscr/rjaf974

**Published:** 2025-12-09

**Authors:** Qusai Belbaisi, Hamza M A Ghaith, Osama N M Atawneh, Nada Ishti, Khadeeja Nofal, Sami Bannoura, Mohammad Y Al-Qadi

**Affiliations:** Faculty of Medicine, Palestine Polytechnic University, Dahiat Al-Baladiya, Hebron, Hebron Governorate, P706, Palestine; Faculty of Medicine, Palestine Polytechnic University, Dahiat Al-Baladiya, Hebron, Hebron Governorate, P706, Palestine; Faculty of Medicine, Palestine Polytechnic University, Dahiat Al-Baladiya, Hebron, Hebron Governorate, P706, Palestine; Faculty of Medicine, Palestine Polytechnic University, Dahiat Al-Baladiya, Hebron, Hebron Governorate, P706, Palestine; Faculty of Medicine, Palestine Polytechnic University, Dahiat Al-Baladiya, Hebron, Hebron Governorate, P706, Palestine; Department of Pathology, Al-Makassed Islamic Charitable Society Hospital, Ruba el-Adawiya St (Mount of Olives / At-Tur), PO Box 19482, Jerusalem; Faculty of Medicine, Palestine Polytechnic University, Dahiat Al-Baladiya, Hebron, Hebron Governorate, P706, Palestine

**Keywords:** leiomyosarcoma, small bowel neoplasm, gastrointestinal sarcoma, DOG1, surgical resection

## Abstract

Small bowel leiomyosarcoma (LMS) is a rare and aggressive malignancy that often presents with nonspecific abdominal symptoms, leading to delayed diagnosis and poor prognosis. We report the case of a 73-year-old male with an eight-month history of abdominal pain, constipation and anemia. Computed tomography revealed partial small bowel obstruction caused by a mass, and exploratory laparotomy identified an ulcerated lesion involving the small bowel and mesentery. The mass was completely resected with primary anastomosis. Histopathological examination confirmed a high-grade LMS with a high mitotic index, positive for smooth muscle actin and desmin, and negative for CD34, S100, and CD117. Notably, weak DOG1 positivity was observed, a rare finding that may complicate distinction from gastrointestinal stromal tumours. This case highlights the diagnostic challenges of small bowel LMS, the central role of surgical resection in management, and the need for careful pathological evaluation to avoid misclassification and guide prognosis.

## Introduction

Small bowel tumours are rare, accounting for <2% of primary gastrointestinal neoplasms. Sarcomas comprise only a minor fraction, with adenocarcinomas more common. Leiomyosarcoma (LMS), a subtype of soft-tissue sarcoma, accounts for ~1% of malignant mesenchymal GI lesions, making it extremely rare [1]. LMS commonly arises in the uterus, GI tract, and retroperitoneum; within the GI tract, the stomach is most frequent, followed by small intestine, colon, and rectum [2]. In the small bowel, LMS occurs most often in the jejunum (32%), ileum (25.2%), and least in the duodenum (12.6%), usually presenting in the 6th decade [3]. Symptoms are nonspecific, leading to delayed diagnosis and poor prognosis [2]. Presentations include intra-abdominal mass, acute abdomen, or GI bleeding. Surgical resection remains the standard of care [4].

## Case presentation

A 73-year-old male presented to the emergency department (ED) complaining of abdominal pain progressively worsening in the past 8 months, associated with episodes of constipation and intermittent vomiting. There was no history of diarrheoa, haematemesis, or melena. Past medical and surgical history was unremarkable, and there were no reported comorbidities.

In the ED his vitals were normal except for a blood pressure 155/85. Abdominal examination revealed mild diffuse tenderness without distension or peritoneal signs. Laboratory investigations on admission were within normal limits except for anemia (hemoglobin 9.2 g/dl).

**Figure 1:**
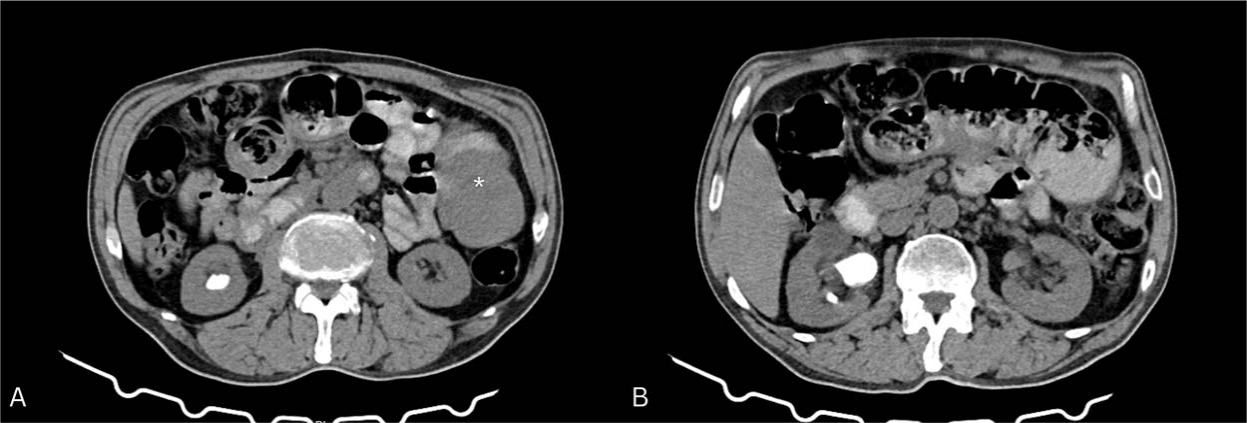
Contrast-enhanced CT of the abdomen. (A) Axial view showing a soft tissue mass-like lesion in the left abdomen (star). (B) Dilated bowel loops with mild to moderate thickening of the bowel wall.

CT abdomen (Fig. 1) showed dilated ileal loops with mild–moderate wall thickening up to 5.7 cm and a 6 cm left-sided soft tissue mass, suggestive of partial small bowel obstruction secondary to neoplasm.

During hospitalization, the patient’s condition remained stable. He passed stools intermittently, and his abdominal symptoms improved with supportive management (intravenous fluids 2500 CC NS 24 HR, antibiotics, and analgesia Pantoprazole 40 MG IV 1^*^2, paracetamol 1MG IV 1^*^3, ciprofloxacin 200 MG IV 1^*^2, Metronidazole 500 MG IV 1^*^3.

**Figure 2:**
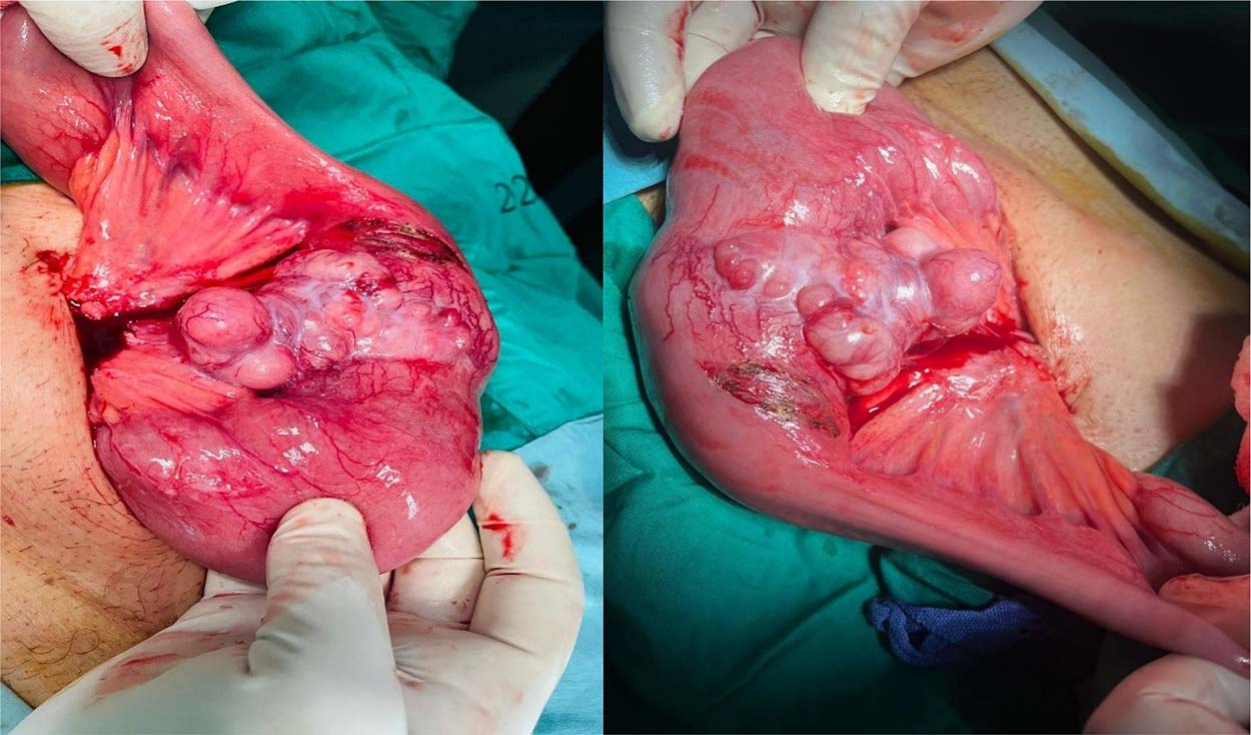
Intraoperative image showing the affected small bowel and mesentery with distended bowel proximal to the mass.

The patient underwent exploratory laparotomy. A 40 × 4 cm small bowel segment with 15 × 3 × 1 cm mesentery was resected (Fig. 2). An 8 × 5 × 6.5 cm ulcerated mass involved the bowel wall and part of mesentery. Primary end-to-end anastomosis was performed. Postoperative recovery was uneventful, and the patient was discharged for oncologic follow-up.

**Figure 3:**
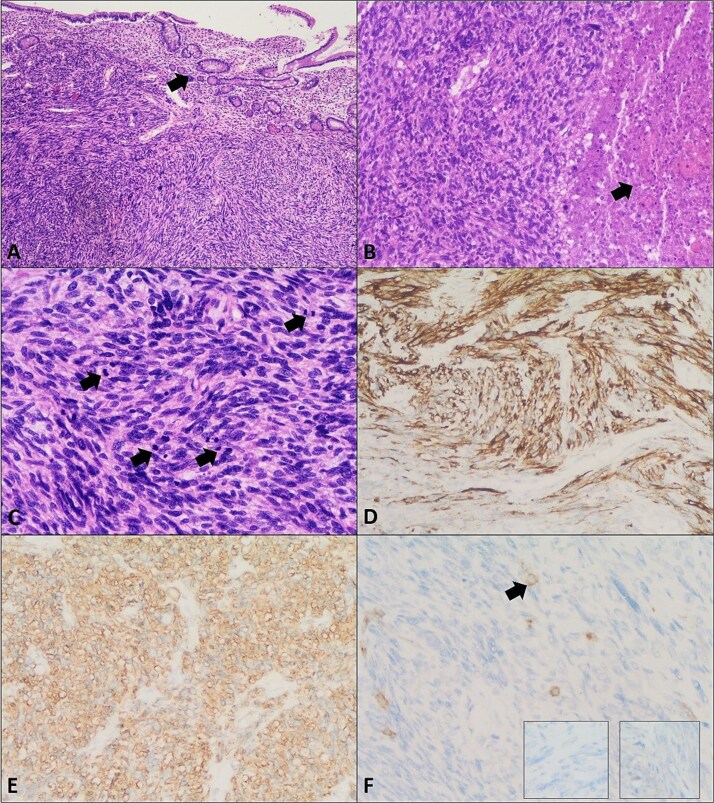
Leiomyosarcoma of the small bowel. (A) Section shows a malignant spindle cell neoplasm composed of intersecting fascicles with overlying small bowel mucosa (arrow) (H&E, ×4). (B) Areas of tumour necrosis are seen (arrow) (H&E, ×10). (C) Increased mitotic activity is noted (arrows) (H&E, ×20). (D) Immunohistochemistry showing positive Desmin. (E) Smooth muscle actin (SMA) immunostain positivity. (F) Negative CD117 (c-kit), Dog-1 (right insert), and S100 (left insert) immunostains confirming the diagnosis of leiomyosarcoma. Arrow in panel F highlights mast cells as a positive internal control for CD117 immunostain.

Histopathology revealed a high-grade spindle cell tumour consistent with LMS, high mitotic rate (~24/10 HPFs), extending into mesentery (pT2b, pN0, and pMx). Immunohistochemistry: positive SMA and desmin (DS), patchy weak DOG1, negative CD34, S100, CD117, and myogenin (Fig. 3).

## Discussion

Small bowel LMS is rare and aggressive, representing >0.06% of GI cancers [5, 6]. Non-specific symptoms like abdominal pain, anemia, and vomiting often delay diagnosis [6]. Upper endoscopy and colonoscopy are limited; CT colonography and MR enterography aid diagnosis [7]. Most patients are male (2:1), presenting in the 6th–8th decade, with ileum the most frequent site [3]. the majority of reported patients present in their sixth to eighth decade of life, with a 2:1 male predominance and a mean age of 69.8 years [3], The main weaknesses of our work is the short follow-up period and the performance of a CT scan instead of a CT Colonoscopy scan.

Table 1, adapted from Mohammed *et al.* (2025), summarizes published cases and integrates both their case and ours for comparison [3], consistent with our patient’s presentation. The typical immunohistochemical profile is positivity for smooth muscle actin (SMA) and DS with negativity for CD117, CD34, and DOG1, which helps distinguish LMS from GISTs. Interestingly, our case differed by demonstrating weak DOG1 positivity, a finding not previously emphasized in other reports, highlighting diagnostic challenges distinguishing LMS from GIST [3].

**Table 1:** Summary of small intestinal leiomyosarcoma: case characteristics and outcomes

**Authors, year**	**Study design**	**No. of cases**		**Age/Sex**	**Clinical presentation**	**Medical history**	**Surgical history**	**Diagnostic method**	**Immunohistochemistry (+), (−)**					**Tumour site**	**Co-occurrence**	**Treatment**	**Outcome and follow-up**
									**CD117**	**CD34**	**DOG1**	**SMA**	**DS**				
Our case	Case report	1		73y/M	Abdominal pain (8 months), constipation, intermittent vomiting, anemia (Hb 9.2 g/dL)	None	None	CT, explor-atory laparotomy, Histo, and IHC	(−)	(−)	(+)^*^	(+)	(+)	Ileum with mesenteric involvement	None	Small bowel resection with end-to-end anasto-mosis	Alive, discharged, referred to oncology for follow-up
Mohammed *et al.*, 2025 [3]	Case report	1		72y/F	Chronic paroxysmal abdominal pain (5 months), abdominal distension with large paraumbilical swelling	HTN, DM, hyperthyroidism	Paraum-bilical hernia repair, prior abd-ominal surgery (bowel-related)	US, endoscopy, colonoscopy, CT, histopathology, IHC	(−)	(−)	(−)	(+)	(+)	Ileum (mesenteric border)	None	Segmental small bowel resection with 15 cm margins	Alive, well at 4 months, no recurrence
Pilipović-Grubor *et al.,* 2023 [5]	Case report	1		55y/F	Abdominal pain, nausea, vomiting, loss of appetite, and diarrhea	Radiation for endometrial cancer and high ovarian tumour markers	Pelvic surgery for endometrial cancer	Ultrasound, X-ray, CT, MRE, Histo, and IHC	(−)	(−)	(−)	(+)	(+)	Ileum	Mesenteric involve-ment	Partial bowel resection with ileo-ileal anastomosis	NA
Bouassida *et al.* 2022 [8]	Case report	1		65y/M	Paroxysmal abdominal pain	None	None	Colonoscopy, CT, MRI, Histo, and IHC	(−)	NA	(−)	NA	(+)	Ileum	None	Surgical resection	Alive with no recurrence at 1 year
Zhou *et al,* 2024 [9]	Case report	2	Case 1	70y/M	Abdominal pain and palpable mass	Hypertension and depression	None	CECT, Histo, and IHC	(−)	(−)	(−)	(+)	(+)	Terminal ileum	None	Right hemicolectomy	Died of infectious shock within 9 months
			Case 2	66y/M	Abdominal pain and diarrhea	Invasive lung adenocarc-inoma	Radical surgery for lung cancer	CECT, Histo, and IHC	(−)	(−)	(−)	(+)	(+)	Duodenum	None	Segmental duodenal resection	No recurrence after a 7-month follow-up.
Ferrari *et al.* 2020 [10]	Case series	4	Case 1	83y/F	Bowel obstruction and chronic abdominal pain	Arterial hypertension	Cholecyst-ectomy for cholelithi-asis	CT, exploratory laparoscopy, Histo, and IHC	(−)	(−)	NA	(+)	(+)	Jejunum	Lymphadenopathy of the mesentery	Jejunal resection and palliative care	Died after a few days
			Case 2	86y/M	Abdominal discomfort and sub-obstruction	NA	None	Ultrasound, CT, percutaneous biopsy, Histo, and IHC	(−)	(−)	NA	(+)	(+)	Ileum	Mesenteric root infiltration and lung metastasis	Trabectedin	Died after 11 months (ischemic stroke)
			Case 3	79y/F	Obstructive mass	NA	Ileal resection	CT, Histo, and IHC	(−)	(−)	NA	(+)	(+)	Ileum	Severe adhesions, colon and rectus muscle infiltration, and postoperative abscess	Ileal resection	Alive with no evidence of recurrence
			Case 4	69y/M	Acute peritonitis and bowel obstruction	Type II diabetes mellitus and chronic kidney disease	Anterior rectal resection for adenoc-arcinoma	CT, Histo, and IHC	(−)	(−)	NA	(+)	(+)	Ileum	Infiltration of the cecum and abdominal wall	Ileal resection	Alive with no evidence of recurrence at 12 months
Niraj and Richards 2021 [11]	Case report	1		45y/F	Chronic abdominal pain	Gastritis and iron deficiency anemia	None	Endoscopy, CT, and UGN	(−)	NA	NA	(+)	(+)	Small intestine (non-specified site)	None	Ultrasound-guided trigger point injection	Discharged on day 5; high-grade LMS excised.
Kim *et al.* 2020 [12]	Case report	1		80y/M	Abdominal pain, palpable mass	Non-small cell lung cancer	Ileocecal resection	CT, biopsy, Histo, and IHC	(+)^*^	NA	(−)	(+)	(−)	Ileum	Brain metastasis	Surgical resection	Died after 3 months
Mazzotta *et al.* 2020 [13]	Case report	1		90y/M	Abdominal pain, nausea, and occlusion.	Hypertension and dyslipidemia	Inguinal hernia repair and hemorrhoidectomy	CT, X-ray colonoscopy, and MRI	(−)	(−)	(−)	(+)	(+)	Ileum	Ischemic bowel and mesenteric lymphadenopathy	Ileocecal resection	No complications and no further treatment.
Wilt *et al.* 2024 [14]	Case report	1		53y/M	Abdominal pain, nausea, and vomiting	DVT, gout, and type 2 diabetes	Right nephrectomy	CT, X-ray, Histo, and IHC	(−)	(−)	(−)	(+)	(+)	Terminal ileum	Adherence to the peritoneum, bladder, and sigmoid colon.	Surgical resection	Local recurrence within 8 weeks
Abou El Joud and Abbasi 2021 [15]	Case report	1		67y/M	Abdominal bloating, weight loss, and varicose veins	Untreated	None	Ultrasound, CT, biopsy, Histo, and IHC	(−)	NA	(−)	(+)	(+)	Small bowel (non-specified site)	Lung and liver metastasis and vena cava compression	Palliative care	Died within 2 months
						hepatitis C											

Y, year. M, male. F, female. NA, non-available. DVT, deep vein thrombosis. CT, computed tomography. PET, positron emission tomography. Histo, histology. IHC, immunohistochemistry. MRE, magnetic resonance elastography. MRI, magnetic resonance imaging. CECT, contrast-enhanced computed tomography. SMA, smooth muscle actin. DS, desmin. (−), negative. (+), positive. (+)^*^, weak positive. UGN, ultrasound-guided needling.

When comparing clinical presentations, most cases—including those by Pilipović-Grubor *et al.* [5], Bouassida *et al*. [8], and Zhou *et al*. [9]—described abdominal pain as the leading symptom. Obstructive features such as vomiting, diarrhea, or palpable masses were also common. Our patient’s 8-month history of abdominal pain with constipation and anemia is in line with this pattern but highlights the protracted and subtle course that can precede diagnosis. Unique presentations were also documented: Niraj and Richards [11] reported misattribution of symptoms to abdominal myofascial pain syndrome, while Abou El Joud and Abbasi [15] noted varicose veins secondary to vena cava compression by metastatic disease.

Treatment across reports was primarily surgical resection, which remains the only potentially curative option. Clear margins were emphasized as essential to reduce recurrence risk [8, 9]. In our case, a segmental resection with end-to-end anastomosis achieved grossly complete excision. Several studies documented poor outcomes despite surgery, highlighting the aggressiveness of LMS. Ferrari *et al.* [10] reported two deaths within a year due to disease progression or complications, and Zhou *et al*. [9] noted mortality from infectious complications within 9 months. Similarly, Kim *et al*. [12] described rapid progression with brain metastasis and death within 3 months. Conversely, Bouassida *et al*. [8] and Mazzotta *et al*. [13] reported patients remaining disease-free at one year or beyond. Our patient remains alive and stable at early follow-up, reflecting the variability of prognosis even with similar management.

Mesenteric involvement, as seen in our case and in reports by Ferrari *et al*. [10], may contribute to more complex surgical resections and could impact recurrence risk. The prognostic significance of such local extensions remains uncertain but warrants attention. Moreover, the heterogeneity of outcomes across the reviewed cases suggests that factors beyond surgical technique—such as tumour biology, grade, and molecular characteristics—likely influence survival.

In summary, small bowel LMS is a diagnostic and therapeutic challenge. Comparison of published cases, including those summarized in Table 1, emphasizes common features such as ileal predilection, SMA/DS positivity, and the central role of surgical resection. Our case adds to the literature by demonstrating weak DOG1 positivity and prolonged nonspecific symptoms prior to diagnosis. These findings reinforce the need for heightened clinical suspicion, careful pathological assessment, and long-term follow-up given the risk of recurrence and metastasis.

## Conclusion

Small bowel LMS is rare and aggressive, often presenting with vague symptoms. Early recognition in elderly patients with chronic abdominal complaints is crucial. Complete surgical resection remains the cornerstone of treatment, with histopathology confirming diagnosis and grade. Reporting rare cases adds valuable insight for earlier recognition and management.
